# Effects of Wash Protocol and Contamination Level on Concentrations of Cortisol and Dehydroepiandrosterone (DHEA) in Swine Hair

**DOI:** 10.3390/ani11113104

**Published:** 2021-10-30

**Authors:** Darian S. Pollock, David M. Janz, Diego Moya, Yolande M. Seddon

**Affiliations:** 1 Department of Large Animal Clinical Sciences, Western College of Veterinary Medicine, University of Saskatchewan, Saskatoon, SK S7N 5B4, Canada; diego.moya@usask.ca (D.M.); yolande.seddon@usask.ca (Y.M.S.); 2 Department of Veterinary Biomedical Sciences, Western College of Veterinary Medicine, University of Saskatchewan, Saskatoon, SK S7N 5B3, Canada; david.janz@usask.ca

**Keywords:** cortisol, dehydroepiandrosterone, hair contamination, swine, wash protocol, stress

## Abstract

**Simple Summary:**

Quantifying the hormones cortisol and dehydroepiandrosterone (DHEA) in swine hair is of increasing interest to evaluate long-term stress and resilience. Because swine hair is often contaminated with varying amounts of excrement, it needs to be decontaminated with a solvent prior to hair hormone extraction to rid the hair of potential external contaminants. However, it is unknown how contamination influences hair hormone concentrations, and if current wash protocols are effective in removing contamination. The goals of this study were thus, to determine if wash solvents (methanol versus isopropanol), contamination level (none, mild, or severe), and the number of washes (one, three, or five) influenced hair cortisol and DHEA concentrations. This study showed that hair cortisol, but not DHEA concentrations were reduced when external contamination was present, and that methanol was more effective at removing external contamination compared to isopropanol. There were also decreasing concentrations of cortisol and DHEA within the hair and wash solvent with an increasing number of washes. Thus, it is recommended not to use contaminated hair for hormone analysis, and to wash swine hair with a minimum of three 3 min methanol washes prior to analysis.

**Abstract:**

The effect of washing procedure and contamination level on the concentrations of cortisol and dehydroepiandrosterone (DHEA) in swine hair was explored over two studies. Hair shaved from finisher pigs (*n* = 8) and sows (*n* = 8, cortisol study 1 only) was split into two treatments (two hair samples/pig) to receive either three isopropanol or methanol washes, and two paired subsamples of hair were contaminated with feces and urine, mildly or severely. Samples were further subdivided and received one, three, or five methanol washes. Hormone concentrations were quantified from the hair and wash solvent, and the ratio of hormones in the solvent to that in the hair calculated. When grouping sow and grower hair together for analysis, hair cortisol concentrations were 13% greater after three isopropanol washes compared to methanol (22.84 ± 3.12 vs. 19.77 ± 2.64 pg/mg, respectively). When analyzing sow and grower hair separately, sow hair cortisol concentrations were 20% higher following three isopropanol washes compared to methanol washes (22.06 ± 5.21 vs. 27.72 ± 5.65 pg/mg), with no differences in grower pig hair concentrations. The solvent cortisol concentrations did not differ with wash solvent. No differences were seen for DHEA. Contamination level did not influence hormone concentrations. Hair cortisol concentrations were 24% higher after one wash compared to five washes (11.98 ± 1.47 vs. 9.05 ± 0.92 pg/mg), whereas the solvent cortisol concentrations were 80% and 84% higher after one wash compared to three and five washes, respectively (21.09 ± 4.04 vs. 4.21 ± 1.62 vs. 3.36 ± 1.32 pg/mg). The solvent–hair cortisol ratio was 65% and 73% higher following one wash compared to three and five washes (1.36 ± 0.80 vs. 0.47 ± 0.12 vs. 0.37 ± 0.14). Hair DHEA concentrations were 39% higher after one wash compared to five washes (42.39 ± 6.87 vs. 26.02 ± 5.69 pg/mg). The solvent DHEA concentrations, and the solvent–hair ratio for DHEA were 94% and 98% and 92% and 98% higher going from one wash to three and five washes, respectively (solvent: 5.07 ± 0.26 vs. 0.28 ± 0.12 vs. 0.12 ± 0.09 pg/mg and solvent–hair ratio: 0.13 ± 0.006 vs. 0.010 ± 0.004 vs. 0.003 ± 0.002). Following three methanol washes, the non-contaminated hair had 46% and 48% higher hair (17.47 ± 1.12 vs. 9.35 ± 0.80 vs. 9.05 ± 1.06 pg/mg) and a 76% and 72% higher solvent (16.31 ± 8.07 vs. 3.92 ± 0.50 vs. 4.50 ± 2.31 pg/mg) cortisol concentration compared to mild and severely contaminated hair, respectively. Wash solvent influences cortisol concentrations in swine hair, but not DHEA. Contaminated swine hair should be avoided in analyses when possible.

## 1. Introduction

Cortisol is a glucocorticoid commonly used as a biomarker of animal welfare due to its release by the hypothalamic pituitary adrenal (HPA) axis in times of stress. However, it has also been shown to increase and/or vary for other reasons such as during feeding [1] and throughout pregnancy [2]. Dehydroepiandrosterone (DHEA) is another hormone of the HPA axis, with roles largely opposing those of cortisol [3]. In humans, higher levels of DHEA have been suggested to be indicative of increased resilience and wellbeing, with decreases linked to a variety of mental and physical conditions, including chronic stress [4]. There is interest to explore the measurement of these hormones in unison as more a sensitive measure of HPA axis activity and, furthermore, to understand whether evaluating the ratio of these hormones could provide a measure of stress and resilience [5]. The measurement of cortisol and DHEA together could provide a sensitive biomarker for animal welfare and to evaluate animal production performance. 

The majority of studies in swine have measured cortisol as a biomarker of short-term stress [6,7,8,9]. When measured in blood or saliva, increased cortisol is believed to be suggestive of acute stress, and in swine, an increase in cortisol is observed following acutely stressful events such as weaning [6], transport [7], mixing [8], and castration [9]. DHEA is a relatively new hormone being measured in pigs, with only a few studies existing [10,11,12,13].

Cortisol [14] and DHEA [15] can be measured in a variety of matrices including saliva, urine, blood, feces, and hair. In contrast to the aforementioned matrices, which provide information on short-term states, hormones within the hair shaft provide information on hormone levels over longer periods of time. Measurement of cortisol and DHEA in hair is of interest for the measurement of chronic stress and offering a method for longer-term assessments of welfare. While the exact mechanisms via which hormones integrate into the hair shaft are not fully understood, four potential mechanisms have been proposed: (1) hormones from the blood passively diffuse into the hair follicle throughout hair growth, (2) hormones released from sebaceous glands integrate into the hair shaft [16], and (3) external contaminations containing hormones incorporate into the hair shaft [17], or (4) a peripheral HPA-like system in hair follicles, melanocytes, epidermal melanocytes, and dermal fibroblasts from which hormones become incorporated into the hair [18]. While the exact mechanisms are unknown, it is likely that steroid hormones are incorporated into hair using all of the mechanisms to varying extents.

Prior to extracting hormones from within the hair, washing is performed to decontaminate the hair surface of external contaminants [19]. To date, all published works in pig hair have used isopropanol as a washing solvent [2,10,11,20,21,22,23,24] following methods recommended by Davenport et al. [25] in rhesus macaque (*Macaca mulatta*) hair. Davenport et al. [25] determined that methanol, but not isopropanol, extracted cortisol from within the hair shaft following three 3 min washes and, thus, concluded that two 3 min isopropanol washes was an ideal washing protocol. However, Kroshko et al. [26] found that bear hair did not show evidence of cortisol leaching from the hair shaft, even after eight 3 min methanol washes. Thus, it is possible that wash solvents behave differently between species, but this has not been investigated in pig.

Pig hair is often contaminated with varying amounts of urine and feces (personal observation). It is also known that different housing environments impact swine hygiene [27]. Thus, in order to accurately use hair analysis as a chronic assessment of HPA axis activity in swine, one must consider other factors contributing to hair glucocorticoid levels, such as external contamination. One study to date has evaluated the effect of external contamination in pig hair and found that the type of contamination (urine, water, or a mixture of water and feces) had different effects on hair cortisol concentrations following two isopropanol washes [24]. Specifically, urine increased hair cortisol concentrations, whereas water and a mixture of water and feces decreased concentrations. However, one limitation to this study is that it did not assess the quantity of hormone washed off from the wash solvents; thus, the efficacy of two isopropanol washes is unknown.

The ratio of the hormone concentration in the solvent to that extracted from within the hair (the solvent–hair ratio) has been proposed as a method of identifying contaminated samples and to identify the efficacy of the washing protocol [28]. Specifically, it has been proposed that when the wash solvent–hair ratio is less than 0.1, the sample is not contaminated, whereas, when the ratio is greater than 0.5, it is externally contaminated, with levels between 0.1 and 0.5 being uncertain. While the solvent–hair ratio was developed for use in assessing the presence of external contamination and efficacy of hair decontamination for drug analysis [28], it may also be beneficial for standardizing hormone analysis.

Three studies to date have examined DHEA concentrations in pig hair [10,11,13]. However, none of the studies validated laboratory methods for DHEA quantification. Enzyme-linked immunosorbent assays (ELISAs) are a common and efficient way to analyze hormone concentrations. These assays, however, require validation for each species to ensure the tests are accurate, sensitive, and precise for the species of interest. Thus, in order to continue researching DHEA and its potential as a biomarker of welfare in swine, it is necessary for validation work to be completed.

The objectives of this research were to (1) determine whether isopropanol versus methanol as hair wash solvents influences the quantity of cortisol and DHEA extracted from pig hair, (2) identify the efficiency of increasing decontamination washes (one, three, and five washes) to remove mild and severe fecal and urine contamination from pig hair, as well as how the level of contamination affects the quantity of hormones measured from the hair, and (3) validate a commercially available DHEA salivary ELISA kit for quantification of DHEA in pig hair and saliva.

## 2. Materials and Methods

Animal Care: All experimental procedures were reviewed and approved by the University of Saskatchewan Animal Care and Use Committee, which is regulated by the Canadian Council on Animal Care. This study was conducted at Prairie Swine Centre, Saskatoon, Canada from October–December 2019.

Animals and Sample Collection: Eight grower pigs (111.30 ± 2.72 kg (mean ± SD)) from the same production batch were used in this study. Pigs were housed in groups of six in fully slatted, barren pens (2.41 m by 1.75 m). Two pens were selected from within the same room, with four pigs per pen being sampled. Pigs were chosen on the basis of gender (*n* = 4 gilts, and *n* = 4 barrows) and cleanliness. All pigs were of the same age, breed (Large White × Landrace) and hair colour (white). Grower pigs were given food and water ad libitum. One feeder and one nipple drinker were provided per pen. Eight excess sow hair samples from another study at the same barn were also used. Sows were raised in fully slatted, barren environments throughout gestation. Water was given ad libitum, with a standard gestation diet quantity provided once daily. Sows were part of another study and were part of three different treatment groups: (1) stall-housed (*n* = 2), (2) stall housed sows that were exercised (walked around the gestation room) for 10 min weekly (*n* = 2), and (3) group-housed sows (*n* = 3). Stalls were 0.61 m × 2.13 m, whereas the group-housed sows were in pens measuring 4.87 m × 3.05 m with four sows per pen. Sows were all of the genetic line, Camborough 42, had an average parity of 1.86 ± 0.32 (range: 0–3), and had an average body condition score of 2.71 ± 0.76.

Growing pigs were walked into a weigh crate and saliva was collected using a pure cotton rope bridle, allowing the pig to chew for 2 minutes. Subsequently, the pig had a 15 × 15 cm^2^ area of hair (approximately 900 mg) shaved from the right side of their rump using electric clippers. The hair was collected on tinfoil and placed into an envelope. Between sample collections of individual pigs, clippers were sprayed with Clippercide^®^ (Barbicide, Milwaukee, WI, USA) and cleaned with a toothbrush to remove any remaining hairs. The sow samples were collected in the same manner, following hair regrowth over one gestation. Saliva was collected from the ropes by centrifugation following methods described by Seddon et al. [29] and were stored at −80 °C until analysis.

### 2.1. Study 1: The Effect of Wash Solvent on Hair and Solvent Cortisol and DHEA Concentrations

**Figure 1:**
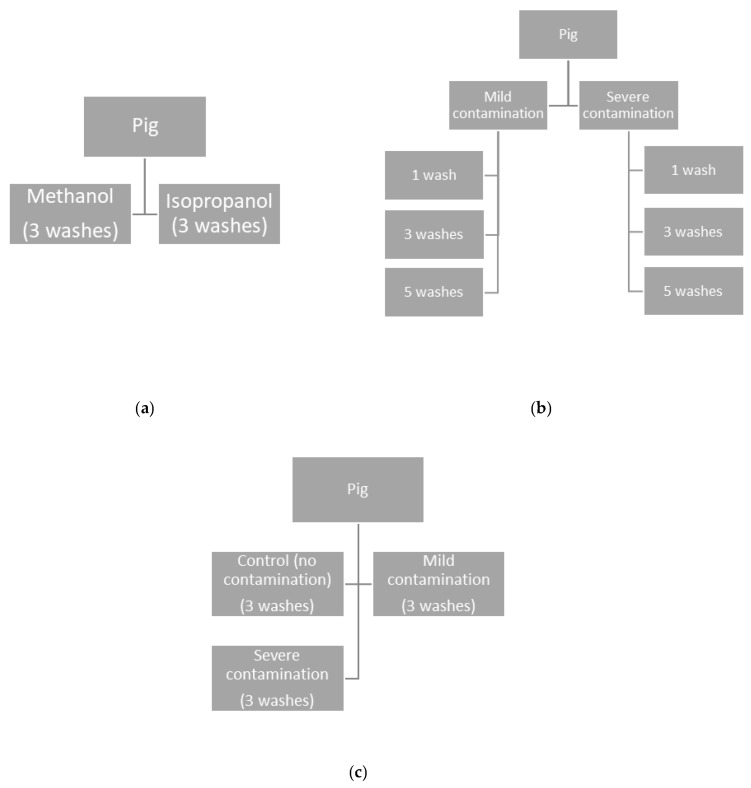
Experimental design of studies performed. Study 1: the effect of wash solvent on hair and solvent cortisol and DHEA concentrations (**a**); Study 2: the effect of contamination level and wash number on hair and methanol solvent cortisol and DHEA concentrations and the solvent-hair ratio (**b**,**c**).

Of the original 900 mg sample of hair collection, two 100 mg subsamples of hair per grow-finisher pig and sow were weighed and split into two treatment groups, creating paired hair samples from the same animal (Figure 1a). Per animal, these paired hair samples each received decontamination washes with either isopropanol or methanol. Hair hormone extractions followed methods by Macbeth et al. [18]. Briefly, 100 mg of hair was weighed and washed in 0.04 mL of solvent/mg of hair for 3 min on a slow rotator. Hair was removed, placed on paper towel, patted dry, and washed twice more, using the same process. Hair was then dried for 24 h at room temperature, the damaged and split ends were trimmed off, and the hair was ground using a Retsch MM 301 Mixer Mill at approximately 0.03 min/mg of hair at 30 Hz, until a fine powder was achieved. Twenty-five milligrams of powdered hair (per hormone measured) was incubated in 0.5 mL of methanol (per hormone measured) for 24 h on a slow rotator and centrifuged (15 min at 2150× *g* at 20 °C), and the supernatants were evaporated at 38 °C under nitrogen gas. The samples were rinsed with 1.5 mL of either isopropanol or methanol, vortexed, and centrifuged; this was done twice, for a total of three pooled supernatants. The tubes were rinsed with decreasing volumes of solvent (0.8, 0.6, and 0.4 mL), evaporated under nitrogen gas, and reconstituted with 0.6 mL of assay diluent buffer (12 h at 4 °C) provided with the ELISA kits. Hormones in the wash solvents were also quantified. This was performed by evaporating the wash solvent under nitrogen gas, with reconstitution as described above, following methods from Kroshko et al. [26]. The reconstituted samples were used to analyze hormone concentrations of cortisol and DHEA in the grower pigs, and cortisol in the sows (due to smaller amounts of hair available).

### 2.2. Study 2: The Effect of Contamination and Wash Number on Hair and Methanol Wash Solvent Cortisol and DHEA Concentrations, and Their Ratio

Using remaining hair from the original 900 mg collected from the same eight grow-finisher pigs as described above, two 300 mg subsamples of hair per pig were used and split into treatment groups: either 25% (mild) or 75% (severe) of the hair surface contaminated with a mixture of feces and urine. The 300 mg of hair treatment groups were further subdivided into three groups of 100 mg, receiving one, three, or five methanol decontamination washes (Figure 1b). Thus, each individual pig served as its own control, with six different treatments (mild contamination with one wash, mild contamination with three washes, mild contamination with five washes, severe contamination with one wash, severe contamination with three washes, and severe contamination with five washes). No sow hair samples were used in this study due to financial reasons and limited hair quantities. Fecal matter covered in urine was collected from above the slats (feces sitting on the slats in the dunging area of the pen) from one grow-finisher pen and was refrigerated until used. The same feces and urine mixture was used for all contamination procedures. A procedure was developed to standardize the contamination of hair in the lab, mimicking surface contamination of hair that can be experienced on pigs. Nine 1 cm strips of duct tape were cut and folded such that 3 mm of the sticky side was exposed. Each hair was taken and placed on the sticky side of the duct tape, with the shaved ends stuck to the sticky portion of the tape. Once each hair from a sample was stuck, another piece of duct tape was placed over the initial piece of tape and pressed firmly, ensuring the hair remained in place for the contamination procedure. Preliminary testing revealed that 25% surface contamination required 0.5 mg of feces/mg of hair, while 75% required 1.5 mg of feces/mg of hair. The feces were mixed with a wooden tongue depressor and weighed out for each sample. Cotton applicators were used to spread feces over the hair shaft as uniformly as possible. Hair was left contaminated at room temperature for 72 h. After this time, the contaminated hair was cut off from the tape, as close to the edge as possible. The remainder of the extraction followed methods described above. Given the results of Study 1, methanol was chosen as the wash solvent for the remainder of the studies.

### 2.3. Validation of a DHEA ELISA Kit

Saliva and hair samples were used to validate a commercial DHEA ELISA kit (Salimetrics, LLC, Carlsbad, CA, USA) for use in swine. Remaining ground hair and saliva from the first section of this study were pooled and used for validation. Calculations for validation were conducted as described by Casal et al. [20]. In short, the validation included evaluating parallelism, the limit of detection, intra- and inter-assay coefficients of variation (%CV; SD/mean × 100%), and percentage recovery. Parallelism was evaluated by taking a high concentration sample and diluting it to approximately 700, 280, 112, 45, and 18 pg/mL with assay buffer. These samples were run in triplicate and diluted to create a standard curve which was then compared to the standard curve generated by the standard solutions by comparing the linear regression lines. The limit of detection was calculated by taking the mean optical density of the zero-standard run in triplicate plus two standard deviations. Precision was evaluated by calculating the %CV of one high- and one low-concentration sample. The inter-assay %CV used a high- and low-concentration sample (the low-concentration sample was the same sample diluted to 1:32) run six times over two plates, whereas the intra-assay %CV ran the same sample six times on the same plate. The %CV was calculated by taking the standard deviation divided by the mean and multiplied by 100%. Recovery was evaluated using powdered hair that previously had hormones extracted and spiking the sample with a low and high concentration standard run in triplicate. Calculations for the percent differences were then calculated.

The manufacturer states the cross-reactivity of the antibody for DHEA as follows: DHEA-S = 0.063%, androstenedione = 0.0378%, 17β-estradiol = below detection limit (BDL), estriol = BDL, estrone = BDL, progesterone = BDL, 17α-hydroxyprogesterone = BDL, testosterone = BDL, dihydroxytestosterone = BDL, dianabol = BDL, 11-hydroxytestosterone = BDL, 19-nortestosterone = BDL, cortisol = BDL, aldosterone = BDL, cortisone = BDL, 11-deoxycortisol = BDL, 21-deoxycortisol = BDL, triamincinolone = BDL, corticosterone = BDL, transferrin = BDL. The cross-reactivities for cortisol were as follows: prednisolone = 0.568%, prednisone = BDL, cortisone = 0.130%, 11-deoxycortisol = 0.156%, 21-deoxycortisol = 0.041%, 17α-hydroxyprogesterone = BDL, dexamethasone = 19.2%, triamcinolone = 0.086%, corticosterone = 0.214%, progesterone = 0.015%, 17β-estradiol = BDL, DHEA = BDL, testosterone = 0.006%, transferrin = BDL, and aldosterone = BDL.

### 2.4. Statistical Analysis

All statistical analyses were performed using IBM SPSS Statistics for Windows, version 25 (Armonk, NY, USA: IBM Corp).

#### 2.4.1. The Effect of Wash Solvent on Hair and Solvent Cortisol and DHEA Concentrations

Data were assessed for normality using Kolmogorov–Smirnov (K-S) tests with alpha set to 0.05. The concentration of cortisol (sow hair (*n* = 8) plus grow-finisher hair (*n* = 8)) and DHEA (only grow-finisher hair (*n* = 8)) within the hair shaft and in the wash solvent when washed with isopropanol versus methanol were compared with paired *t*-tests or Wilcoxon signed rank tests, depending on normality of the dataset, to assess differences between paired samples. For data analyzed using paired *t*-tests, the difference between the two treatment groups was assessed for normality. Due to possible variation between sow hair and grow-finisher hair, statistical analysis was initially conducted on both these groups separately, after which it was combined for a final analysis due to no significant differences being found.

#### 2.4.2. The Effect of Contamination and Wash Number on Cortisol and DHEA Concentrations Measured in Hair and Methanol Wash Solvent, and the Solvent–Hair Ratio

One-sample K–S tests were performed to assess normality, and *F* tests were performed for heteroskedasticity. Residual plots were created to visually assess data and identify outliers. All data underwent transformation to meet the assumptions of normality and/or heteroskedasticity. Hair cortisol and DHEA concentrations, and the solvent–hair ratio of cortisol were square root transformed, and the solvent DHEA and the solvent–hair ratio of DHEA values were transformed by taking one plus the logarithm, whereas the solvent cortisol concentration was ranked.

One outlier was removed from the dataset (hair DHEA concentration following five washes of severely contaminated hair for one pig). This outlier had a concentration that was below the detection limit of the ELISA and thus, had a concentration of zero. The same hair had a value of 25.63 following five washes in mildly contaminated hair. This was also the only hair sample that was below the detection limit. It was, thus, concluded that this outlier was a technical error and not a biologically relevant outlier. While other outliers were present, the concentrations were within biologically acceptable limits and were thus, kept in the analyses.

The effect of fecal and urinary contamination level, the number of decontamination washes (one, three, or five), and their interaction was evaluated using a nested ANOVA, with contamination level and the number of washes as fixed factors, and the individual pig as the nested factor (experimental design displayed in Figure 1b). When no interactive effects between factors were present, the interaction was removed from the model. A second analysis was conducted using hair from Study 1. Grow-finisher hair following three methanol washes from Study 1 was used as the control (noncontaminated samples) to compare against mild and severely contaminated hair following three methanol washes. The effects of contamination level following three washes was assessed using a nested ANOVA, where contamination level was a fixed factor, and the individual pig was used as the nest (experimental design shown in Figure 1c). Tukey tests were used for post hoc analysis.

Data were presented as the mean plus or minus the standard error of the mean or as the median with the first and third quartiles of the raw data with the mean or median differences and the 95% CI of the differences. The raw data were run through the final model to provide descriptive statistics and graphs. Statistical results of *t*-tests and Wilcoxon signed rank tests were presented with *t* and *W* respectively, with the subscripts representing the degrees of freedom. Results were presented to indicate the differences in hair hormone concentrations between three washes performed with either isopropanol or methanol. Depending on the analysis, the mean or median values were also presented to provide information on the hormone levels obtained. All ANOVAs were represented with an *F*-value, with the subscripts representing the degrees of freedom within and between treatment groups. The *p*-values represented with the ANOVA analyses represent the fixed effects, and the *p*-values of the Tukey post hoc tests were presented in displaying the comparisons between groups. When no significance was present in the main effects, no post hoc tests were performed. When displaying post hoc analysis data, the difference in means of the transformed data is presented.

## 3. Results

### 3.1. Study 1: The Effect of Wash Solvent on Hair and Solvent Cortisol and DHEA Concentraions

Table 1 contains concentrations of the hair and wash solvent for the gilts and barrows as descriptive statistics, as well as the concentrations and the statistical output for the analysis of growers and sows. There was no significant difference between hair cortisol concentrations after three methanol versus isopropanol washes in grower pigs; however, there was a significant difference for sow hair (*t*_7_ = −2.820, *p* = 0.026) (Table 1). Further analysis revealed no significant differences in hair hormone concentrations between sows and grower pigs for methanol (*t*_7.65_ = −0.860, *p* = 0.404) or isopropanol (*t*_8.23_ = −1.657, *p* = 0.135); therefore, the data was combined to increase the sample size for a final analysis. The mean difference in cortisol concentration between methanol and isopropanol-washed hair was −3.07, with a 13% higher mean cortisol concentration (grower and sow hair combined) after three isopropanol washes compared to three methanol washes (paired *t*-test, *t*_15_ = −2.14, *p* = 0.049, Table 1). There was no effect on hair or solvent DHEA concentrations (Table 1).

### 3.2. Study 2: The Effect of Contamination and Wash Number on Cortisol and DHEA Concentrations Measured in Hair and Methanol Wash Solvent, and the Solvent–Hair Ratio

#### 3.2.1. Cortisol

**Figure 2:**
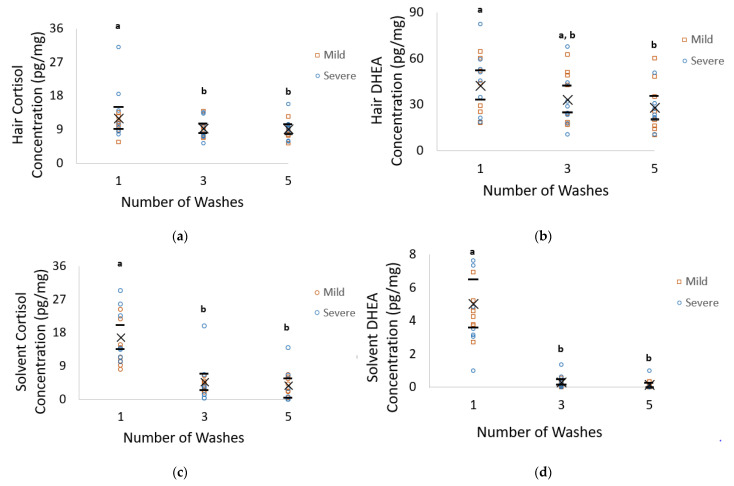

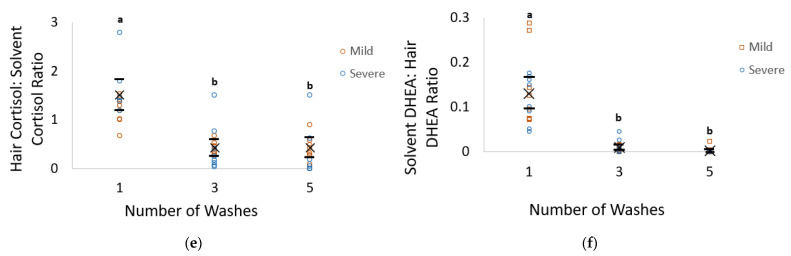
Hair cortisol (**a**) and DHEA (**b**), solvent cortisol (**c**) and DHEA (**d**) concentrations (pg/mg), and the solvent–cortisol (**e**) and solvent–DHEA ratios (**f**) following one, three, and five methanol washes of contaminated grower pig hair (*n* = 16 per treatment). Within a figure graph, different letters denote significant differences between treatment groups (*p* < 0.05), means are represented by ×, and the 95% confidence intervals are represented by black horizontal bars. Significant mean differences in raw data were as follows: hair cortisol (2a): one wash vs. three washes: 2.76 pg/mg, one wash vs. five washes: 2.93 pg/mg; hair DHEA (2b): one wash vs. five washes: 14.62 pg/mg; solvent cortisol (2c): one wash vs. three washes: 16.82 pg/mg, one wash vs. five washes: 17.67 pg/mg; solvent DHEA (2d): one wash vs. three washes: 4.79 pg/mg, one wash vs. five washes: 4.95 pg/mg; solvent cortisol–hair cortisol (2e): one wash vs. three washes: 1.23, one wash vs. five washes: 1.30 solvent DHEA–hair DHEA (2f): one wash vs. three washes: 0.12, one wash vs. five washes: 0.13.

There was an effect of the number of washes on hair (*F*_2,36_ = 4.87, *p* = 0.013) and methanol wash solvent (*F*_2,37_ = 24.00, *p* < 0.001) cortisol concentrations and the solvent–hair ratio of cortisol (*F*_2,37_ = 31.05, *p* < 0.001). The mean hair cortisol concentration after one wash was 24% (a mean difference of 2.76 pg/mg (0.59, 4.93)) and 25% (a mean difference of 2.92 pg/mg (0.90, 4.9)) higher than the concentration after three and five washes respectively (11.98 ± 1.47 vs. 9.22 ± 0.91 and 9.05 ± 0.92 pg/mg, *p* ≤ 0.004; transformed mean difference of 0.09 (0.03, 0.16), 0.10 (0.03, 0.17), Figure 2a). The mean methanol wash solvent cortisol concentration was 80% (a mean difference of 16.82 pg/mg (6.74, 26.89)) and 84% (a mean difference of 17.67 pg/mg (7.59, 27.75)) higher after one wash compared to three and five washes, respectively (21.02 ± 4.04 vs. 4.21 ± 1.62 vs. 3.36 ± 1.32 pg/mg, *p* < 0.001, a transformed mean difference of 20.23 (14.72, 25.74). 23.05 (17.54, 28.55), Figure 2c). The solvent–hair cortisol ratio was 65% (a mean difference of 1.23 (0.76, 1.71)) and 73% (a mean difference of 1.30 (0.80, 1.79)) higher after one wash compared to the ratio at three and five washes (1.65 ± 0.80 vs. 0.47 ± 0.12 vs. 0.37 ± 0.14, *p* ≤ 0.001, a transformed mean difference of 0.57 (0.38, 0.76), and 0.69 (0.50, 0.88), Figure 2e). There was a significant effect of the nest (the individual pig) on hair cortisol concentrations (F_7,36_ = 9.15, *p* < 0.001).

#### 3.2.2. DHEA

Hair DHEA concentrations were affected by the number of washes (*F*_2,37_ = 5.14, *p* = 0.012, Figure 2b), with one wash having a 39% (a mean difference of 14.64 pg/mg (7.03, 25.72)) higher DHEA concentration than five washes (42.39 ± 6.87 vs. 27.75 ± 5.69 pg/mg, respectively, *p* = 0.008, transformed mean difference of 1.24 (0.28, 2.21), Figure 2b). There was also an effect of wash number on methanol wash solvent (*F*_2,37_ = 47.46, *p* < 0.001) and the solvent: hair ratio (*F*_7,37_ = 4.16, *p* = 0.024). The DHEA concentration in the first wash was 94% (a mean difference of 4.81 pg/mg (3.22, 6.35)) and 98% (a mean difference of 4.95 pg/mg (3.93, 6.49)) higher than after three and five washes respectively (5.07 ± 0.26 vs. 0.28 ± 0.12 vs. 0.12 ± 0.09 pg/mg, *p* < 0.001, a transformed mean difference of 1.33 (0.94, 1.72), 1.64 (1.20, 2.09), Figure 2d). The solvent:hair DHEA ratio was 92% (a mean difference of 0.12 (0.08, 0.16)) and 98% (a mean difference of 0.13 (0.10, 0.16)) higher following one wash compared to three and five washes respectively (0.13 ± 0.006 vs. 0.010 ± 0.004 vs. 0.003 ± 0.002, *p* < 0.001, a transformed mean difference of 1.21 (0.89, 1.52), 1.62 (1.25, 1.99), Figure 2f). There was also an interaction between the individual pig and contamination level on hair DHEA concentrations (*F_7_*_,29_ = 2.35, *p* = 0.05).

**Figure 3:**
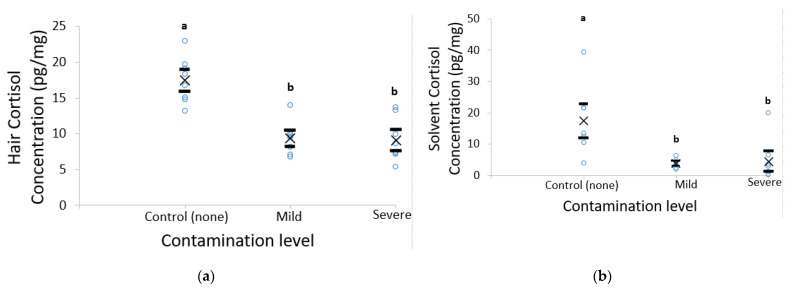
Hair (**a**) and solvent (**b**) cortisol concentrations (pg/mg) of hair receiving no contamination (control) or mild and severe contamination following three methanol washes (*n* = 8 per treatment). Different letters denote significant differences between treatment groups (*p* < 0.05); means are denoted by ×, and 95% confidence intervals are denoted by black horizontal lines.

Hair cortisol concentrations were 46% (a mean difference of 8.12 pg/mg (5.53, 10.71)) and 48% (a mean difference of 8.42 pg/mg (6.04, 10.73)) higher in the control (uncontaminated group) compared to the mild and severely contaminated groups (17.47 ± 1.12 vs. 9.35 ± 0.80 vs. 9.05 ± 1.06 pg/mg, *F*_2,14_ = 33.24, *p* < 0.001, a transformed mean difference of 0.10 (−0.001, 0.20), 0.10 (0.00, 0.20) Figure 3a). The methanol wash solvent cortisol concentrations were also higher in the control compared to the mild and severely contaminated groups (*F*_2,13_ = 8.25, *p* = 0.006), with the control having a 76% (a mean difference of 12.39 pg/mg (5.59, 21.46)) and 72% (a mean difference of 11.81 pg/mg (3.78, 21.27)) higher concentration of cortisol than the mild and severely contaminated hair groups respectively (16.31 ± 8.07 vs. 3.92 ± 0.50 vs. 4.50 ± 2.31 pg/mg, *p* ≤ 0.01,a transformed mean difference of 0.50 (−0.002, 1.19), 0.90 (0.31, 1.50), Figure 3b). No differences were seen regarding the solvent–hair ratio for cortisol, along with any of the DHEA analyses (Table 2).

### 3.3. Validation of a DHEA ELISA Kit

The DHEA ELISA kit was validated for the use in pig hair and saliva. The test for parallelism failed to show unequal slopes in saliva (*F* = 0.048, *p* = 0.833) and hair (*F* = 0.143, *p* = 0.723). The limit of detection was determined to be 6.95 pg/mL, and the percentage recovery was 87.91% and 90.18% for high and low hair DHEA concentrations, respectively. Intra-assay %CV was 10.82% and 6.90% for low DHEA concentration in saliva and hair, respectively, and 5.44% and 10.09% for high DHEA concentration in saliva and hair, respectively. Inter-assay %CV for low DHEA concentration was 12.67% in saliva and 13.66% in hair, and 14.07% and 13.00% for high DHEA concentration in saliva and hair, respectively.

## 4. Discussion

### 4.1. Study 1: The Effect of Wash Solvent on Hair and Solvent Cortisol and DHEA Concentrations

This study aimed to identify whether isopropanol versus methanol as a wash solvent used to decontaminate swine hair impacted the concentrations of cortisol and DHEA extracted from the hair, the solvent, and the solvent–hair ratio. Unlike DHEA, cortisol was affected by the two different wash solvents, with methanol resulting in a lower concentration of cortisol detected in hair following three 3 min washes. The difference between the two solvents, however, was only 13%, and the physiological significance was not clear as it is not currently possible to associate hormone levels in hair with circulating physiologically relevant concentrations. However, it is also important to note that when sow and grower hair was analyzed separately, only sow hair had higher hair cortisol concentrations following three isopropanol washes. However, as there was no significant difference between the concentrations of hair cortisol concentrations between sows and grower, the data were combined. Furthermore, while there were no differences in solvent hormone concentrations following three isopropanol vs. methanol washes when the sow and grower hair was combined, when separate, there were opposing trends. Due to the difference between methanol and isopropanol in sows and when the groups were combined, methanol is suggested as the wash solvent due to the results in the sow hair; moreover, due to the desire to compare hair hormone concentrations between different subgroups and ages of pigs, it may universally offer a better choice of solvent.

Previous work has identified differences between the two wash solvents in rhesus macaque and bear hair. Specifically, Davenport et al. [25] determined that after three 3 min washes, methanol extracted cortisol from within the hair shaft, as indicated by an increase in solvent cortisol concentrations with successive washes. Thus, Davenport et al. [25] recommended two isopropanol washes for rhesus macaque hair. Consequently, the findings of Davenport et al. [25] have been followed for swine hair analysis [9,10,12,19,20,21,22,23,24]. In contrast, Kroshko et al. [26] found that, even after nine 3 min methanol washes, methanol did not extract cortisol from within the hair shaft in bear hair, as indicated by decreases or no changes in the cortisol wash solvent concentrations with increasing washes. Results from Kroshko et al. [26], thus, recommended three methanol washes prior to hair hormone analysis. It is thus possible that the ideal washing protocol is species-dependent; however, this has never been evaluated in swine hair. One possible reason for the difference between the two hormones found in the present study could be due to the differences in chemical structure, which in turn affects their solubility. While cortisol and DHEA share the same cyclopentanophenanthrene ring structure [30], cortisol is a larger molecule with a mass of 362.5 g/mol with an additional ketone group and two additional hydroxyl groups, compared to DHEA with a mass of 288.4 g/mol [31]. However, no other studies have examined the washing dynamics of these two hormones; hence, no studies are available for comparison. Furthermore, considering the knowledge that methanol is a more aggressive wash solvent [32], this could indicate that methanol is more effective in eliminating external contamination; thus, three washes with isopropanol may leave higher amounts of external contaminants remaining on the hair.

The reason for the difference between sows and grower pigs could be due to physical differences in the hair. The visual difference noted was that sow hair was longer and coarser than the grower hair, likely a result of the sows being older. While no studies exist comparing physical hair characteristics in swine or in hair cortisol or DHEA analysis in any species, forensic hair testing for drug analysis has determined that hair pigmentation, keratin content, permeability, moisture content, and hair damage impact the uptake and concentrations of drugs in the hair [33]. It is thus possible that one or more of these physical characteristics could cause certain pigs or groups of pigs to be affected differently by different wash solvents. This information should be regarded when considering a wash solvent, and the results of this study demonstrate the value of examining how the wash solvent affects the hormone concentration in hair.

One limitation to this study was the small amount of sow hair available. For this reason, sow hair was only used to analyze differences in cortisol concentrations between the two solvents. Thus, the sample size for the cortisol group was twice as high (*n* = 16, with *n* = 8 sows and *n* = 8 grow pigs) compared to the DHEA group. However, power analysis (two-tailed significance, with power of 0.8) revealed that a sample size of 374 would be needed to see a significant difference for hair DHEA concentrations; thus, no differences were expected with a sample size of 16.

This is one of three existing studies that measured DHEA levels in swine hair [9,10]. Previous published work showed great variation in measured DHEA levels. Bergamin et al. [10] obtained average values in barrows of 29.51 and 17.64 pg/mg in gilts at 36 weeks of age. In contrast, Trevisan et al. [9] found much higher values, with healthy pigs having levels of 387.7 ± 116.4 pg/mg and pigs infected with *T. solium* at 253.9 ± 82.3 pg/mg. The values found in the present study were closest in number to those seen by Bergamin et al. [10] with clean hair having an average of 36.29 ± 6.16 pg/mg. Studies that exist evaluating hair cortisol concentrations had similar values to those found in the current study. In the present study, DHEA concentrations ranged from 9.05 ± 1.06 pg/mg in contaminated hair to 17.47 ± 1.12 in clean hair. This is within the expected range, as other studies reported values ranging from 4.45 ± 0.335 [10] to 36.23 ± 18.97 [23].

### 4.2. Study 2: The Effect of Contamination and Wash Number on Cortisol and DHEA Concentrations Measured in Hair and Methanol Wash Solvent, and the Solvent–Hair Ratio

The goals of this study were to determine if external contamination of the hair shaft with feces and urine and the number of washes impacted the concentration of hormones extracted from the hair, the solvent, and their ratio. Calculating the ratio of hormone concentrations from the solvent to that extracted within the hair was conducted as a method in evaluating washing efficacy as recommended by Tsanaclis and Wicks [28]. Our results consistently showed an increase in hair and solvent hormone concentrations, as well as their ratio, after one wash compared to five washes, suggesting that the majority of external contamination was removed following one wash. Despite the concentrations of DHEA being on average three times higher than those of cortisol within the hair shaft, the amounts detected in the wash solvent were consistently lower. This suggests that DHEA may be present in smaller amounts in swine feces and urine, or that DHEA may more readily become incorporated into the hair shaft compared to cortisol. However, to the authors’ knowledge, no studies to date are available regarding concentrations of DHEA in swine urine and feces.

Tsanaclis and Wicks [28] determined that taking the ratio of the concentrations of analytes washed off into the solvent to that extracted from within the hair was a valuable method in determining when hair was externally contaminated, as well as to assess the efficacy of the wash protocol. Specifically, ratio values less than 0.1 were suggested to indicate a noncontaminated sample, while values above 0.5 indicate the presence of external contamination, and values between 0.1 and 0.5 are uncertain. Our study found that the solvent–hair ratio for cortisol had a value larger than 0.5 following one wash, with values between 0.1 and 0.5 following three and five washes, suggesting that the hair is still contaminated following one wash, with possible contamination following three and five washes. However, DHEA consistently had values lower than 0.1, suggesting that, even after one wash, external contamination was not present in these samples. It is thus recommended hair be washed with methanol three times to reduce the risk of contamination, particularly for cortisol analysis. One limitation to our study was that the hair hormone concentration after two washes was not analyzed. The majority of pig research used two isopropanol washes [9,10,12,22,23,24], with the remaining studies using an unspecified number of isopropanol washes [20,21]; thus, future research determining differences between three methanol washes and two isopropanol washes would be beneficial for both cortisol and DHEA. While our study suggests that DHEA does not differ between the two solvents, it does clearly indicate a difference in concentrations with increasing wash numbers. Thus, it is possible that whereas the solvent did not affect DHEA concentrations, washing number still had a significant effect.

There was a nest effect for hair and solvent cortisol concentrations, which indicates an effect at the level of the individual pig, in addition to an interactive effect between the individual pig and the level of contamination for hair DHEA. Despite only eight grower pigs being used in this study, each pig was used as its own control to account for possible variations. As stated above, individual hair characteristics are also known to impact drug analysis in human hair; thus, it is likely that it could also affect the uptake of fecal and urinary contamination in swine hair, as well as the efficacy of the wash protocol.

The second study revealed that, after three washes, the uncontaminated group had higher concentrations of cortisol, but not DHEA, in both the hair and the wash solvent compared to the mild and severely contaminated groups, which did not differ. It is possible that this difference was due to hair in the contamination groups being left contaminated in feces and urine for 72 h. Previous studies have shown that water lowers hair cortisol concentrations in pigs [24] and rhesus macaques [34]. It is, thus, possible that moisture caused swelling of the cuticle of the hair leading to leaching of hormones from within the hair shaft. However, Otten et al. [24] reported that urine increased hair cortisol concentrations, suggesting the external cortisol may become incorporated over time. Otten et al. [24] contaminated hair continuously for 4 weeks by submerging hair in liquid contamination and rotating the hair for 2 h. In contrast, our study smeared a mixture of feces and urine on hair and left it for 72 h. This methodology was chosen as it should replicate what would be expected in commercial pigs. We chose this method in order to best replicate the manner of contamination seen in swine hair in the barn. It would be quite unlikely for hair to be completely submerged in urine and feces for 2 h a day for many weeks in a row, particularly for swine in a fully slatted system. Wang et al. [35] found that it takes somewhere between 1 and 5 days for external cortisol contamination (from cortisone cream) to become integrated into the hair shaft of human hair. Thus, it is possible that 72 h was not long enough for the external hormones to become integrated with the hair shaft, but perhaps long enough to damage the cuticle and cause leaching of hormones from within the hair.

Another factor that may have influenced the results is the type of contamination that was used. Otten et al. [24] determined that urine increased hair cortisol concentrations, whereas a mixture of feces and water decreased concentrations. Since water lowers hair cortisol concentrations, it cannot be certain that it was the feces lowering the concentrations in the previously mentioned study, since it was mixed with water. However, given the results from the current study, it is suggestive that this may be the case. While the current study used a mixture of feces and urine, the feces was collected from above the slats in a fully slatted system. For this reason, it is possible that much of the urine fell into the slurry prior to collection, resulting in the majority of the mass used to contaminate hair being feces. One limitation to this study is that the concentrations of the urine and feces mixture were not analyzed due to financial restrictions and no validation methods being available for DHEA in swine feces. Furthermore, had this been measured, it would still not have been possible to identify how much of the external contamination incorporated into the hair shaft. However, other studies have suggested that urine has higher amounts of cortisol compared to feces and hair on a mass/mass basis. Hair cortisol concentrations in this study varied from 5.40–31.07 pg/mg. Carlsson et al. [36] found feces to have a range of approximately 5–45 pg of cortisol per mg of feces. In contrast, Otten et al. [24] found an average urinary cortisol concentration of 324.6 ng/mL, which is the equivalent of 324,500 pg of cortisol per mg of urine. Given these numbers, it is likely that urine has a higher probability of impacting hair hormone analysis. Future research analyzing the difference in urine versus feces in a manner closely resembling real life contamination would be beneficial. It would also be of use to determine if the age of pig has an effect on contamination. Pig hair is quite fine as a piglet compared to when older, e.g., around 60 kg in live weight (personal observation). Furthermore, older pigs have a high percentage of damaged hair, which has shown to increase the integration of external contaminants in human hair externally contaminated with cocaine [37]. Thus, it is possible that older pigs with thicker and more damaged hair incorporate dirt more readily.

### 4.3. Validation of a DHEA ELISA Kit

Despite increasing research being conducted on DHEA as a biomarker of stress and resilience in swine [10,11,12], no laboratory validation of commercially available kits has been conducted for the analysis of DHEA in swine hair or saliva. Validation is a necessary step to ensure future research evaluating DHEA in swine is reliable. The validation procedure in this study included parallelism, inter and intra-assay coefficients of variation, recovery, and the limit of detection for swine hair and saliva. The values obtained were similar to those seen by Casal et al. [20] who validated another commercially available ELISA kit (Salimetrics High-Sensitivity Salivary Cortisol EIA kit) for hair cortisol in swine.

### 4.4. Recommendations

The recommendations based on this study are that, when analyzing swine hair for cortisol and DHEA, the hair should be washed a minimum of three times for 3 min with methanol prior to analysis. It is also recommended that, when possible, the collection of hair contaminated with urine and feces be avoided, and, if used, that it is washed soon after collection to avoid external contaminants becoming incorporated into hair. It should also be noted that, even after five washes, it is not possible to remove all external contamination. Further research is required to determine the length of time at which external contamination becomes incorporated into swine hair, and how this may differ with different contaminants and different physical hair characteristics. As there are differences in physical hair characteristics between subgroups of pigs (such as piglets vs. growers vs. sows), future studies evaluating possible differences in the effects of contamination and wash protocol on each of these groups would also be valuable.

## 5. Conclusions

To our knowledge, this is the first study to evaluate the effect of wash solvent on concentrations of cortisol and DHEA in swine hair, the second study to evaluate the effects of external contamination on hair hormone analysis in pigs, and the first study to evaluate the effects of contamination on hair DHEA analysis, in addition to evaluating the decontamination procedure. Our study suggests that hair contaminated with feces and urine impacts the concentration of cortisol and DHEA extracted from the hair; thus, it is recommended to avoid using contaminated hair when possible. Moreover, it is recommended that three 3 min decontamination washes with methanol be used to effectively rid the hair of contamination. Further studies are necessary to investigate how external contamination is incorporated into the hair shaft, and if the hair-to-solvent ratio is a valid and useful tool in animal hair studies. In addition, evaluating the differences between the standard two 3 min isopropanol washes and the recommended three 3 min methanol washes from the current study would be beneficial.

## Figures and Tables

**Table 1:** Concentrations of cortisol and DHEA (pg/mg) and difference in the hair and wash solvents in pigs separated by gender and animal age. Data expressed as the mean ± SEM or median and first and third quartiles, depending on normality of data.

	Cortisol				
Subgroup of Pig	Matrix	Methanol	Isopropanol	Difference	Statistical Result
Gilts ^∆^	Hair	19.27 ± 1.32	18.76 ± 3.24	n/a	n/a
	Solvent	10.07 ± 2.14	57.04 ± 12.69	n/a	n/a
Barrows ^∆^	Hair	15.67 ± 1.40	17.14 ± 1.71	n/a	n/a
	Solvent	43.72 ± 16.86	43.96 ± 5.21	n/a	n/a
Grower pigs	Hair	17.47 ± 1.12	17.95 ± 3.36	−0.49 (−4.45, 3.37)	*t*_7_ = −0.283, *p* = 0.785
	Solvent ^†^	17.39 (11.01, 34.83)	46.72 (35.06, 58.66)	−21.63 (−55.18, 35.26)	*Z*_7_ = −1.680, *p* = 0.093
Sows	Hair	22.06 ± 5.21 ^a^	27.72 ± 5.65 ^b^	−5.66 (−10.41, −0.91)	*t*_7_ = −2.820, *p* = 0.026
	Solvent ^†^	18.74 (7.73, 52.35)	9.75 (8.25, 15.99)	7.35 (−2.88, 60.80)	*Z*_7_ = −1.680, *p* = 0.093
Sow and grower pigs combined	Hair	22.84 ± 3.12 ^a^	19.77 ± 2.64 ^b^	−3.07 (−6.12, −0.03)	*t*_15_ = −2.143, *p* = 0.049
	Solvent ^†^	18.41 (11.01, 37.32)	26.58 (9.50, 36.72)	−2.12 (−17.95, 22.12)	*Z*_15_ = −0.362, *p* = 0.717
DHEA					
Gilts ^∆^	Hair	39.25 ± 12.43	24.41 ± 6.26	n/a	n/a
	Solvent	0.48 ± 0.27	0.20 ± 0.03	n/a	n/a
Barrows ^∆^	Hair	33.32 ± 4.13	39.71 ± 6.24	n/a	n/a
	Solvent	0.11 ± 0.02	0.11 ± 0.04	n/a	n/a
Grower pigs combined	Hair	36.29 ± 6.16	34.06 ± 4.42	2.23 (−11.56, 15.71)	*t*_7_ = 0.390, *p* = 0.709
	Solvent ^†^	0.11 (0.04, 0.70)	0.15 (0.11, 0.22)	−0.021 (−0.13, 0.72)	*Z*_7_ = −0.420, *p* = 0.674

^a,b^ Different letters within the same row indicates significant differences of *p* < 0.05. Grower pigs (*n* = 4 gilts, *n* = 4 barrows, *n* = 8 total) were 19 weeks of age. Sows (*n* = 8) ranged in parity from 0–3. ^∆^ Denotes no statistical analysis performed on data due to small sample sizes. Differences: mean difference and 95% confidence interval; nonparametric analysis, median difference and distribution free 95% confidence interval. ^†^ Denotes data which underwent nonparametric analysis (Wilcoxon signed rank test); thus, the medians and quartiles are presented.

**Table 2:** Concentrations (pg/mg) of DHEA in the hair and wash solvent of the control (clean), mild, and severely contaminated (mixture of feces and urine) grower swine hair (*n* = 16/treatment) following three 3 min methanol washes. Data are expressed as the mean ± SEM (*n* = 8 per treatment). No statistical differences were present.

					Difference		
Matrix	Control	Mild Contamination	Severe Contamination	Statistical Result	Control vs. Mild Contamination	Control vs. Severe Contamination	Mild vs. Severe Contamination
Hair	36.28 ± 6.16	35.37 ± 6.46	29.23 ± 6.48	*F*_2,13_ = 0.502, *p* = 0.617	0.91	7.05	6.14
Solvent	0.29 ± 0.14	0.22 ± 0.07	0.33 ± 0.16	*F*_2,14_ = 0.036, *p* = 0.964	0.07	−0.04	−0.11

## Data Availability

The data presented in this study are available on request from the corresponding author.

## References

[1] Sapolsky R.M., Romero L.M., Munck A.U. (2000). How Do Glucocorticoids Influence Stress Responses? Integrating Permissive, Suppressive, Stimulatory, and Preparative Actions. Endocr. Rev..

[2] Bacci M.L., Nannoni E., Govoni N., Scorrano F., Zannoni A., Forni M., Martelli G., Sardi L. (2014). Hair cortisol determination in sows in two consecutive reproductive cycles. Reprod. Biol..

[3] Gabai G., Mongillo P., Giaretta E., Marinelli L. (2020). Do Dehydroepiandrosterone (DHEA) and Its Sulfate (DHEAS) Play a Role in the Stress Response in Domestic Animals?. Front. Veter. Sci..

[4] Wolkowitz O.M., Epel E.S., Reus V.I. (2001). Stress hormone-related psychopathology: Pathophysiological and treatment implications. World J. Biol. Psychiatry.

[5] Whitham J.C., Bryant J.L., Miller L.J. (2020). Beyond Glucocorticoids: Integrating Dehydroepiandrosterone (DHEA) into Animal Welfare Research. Animals.

[6] Yang C.-H., Ko H.-L., Salazar L.C., Llonch L., Manteca X., Camerlink I., Llonch P. (2018). Pre-weaning environmental enrichment increases piglets’ object play behaviour on a large scale commercial pig farm. Appl. Anim. Behav. Sci..

[7] Wei S., Xu H., Xia D., Zhao R. (2010). Curcumin attenuates the effects of transport stress on serum cortisol concentration, hippocampal NO production, and BDNF expression in the pig. Domest. Anim. Endocrinol..

[8] Tuchscherer M., Puppe B., Tuchscherer A., Kanitz E. (1998). Effects of social status after mixing on immune, metabolic, and endocrine responses in pigs. Physiol. Behav..

[9] Sutherland M.A., Davis B.L., Brooks T.A., McGlone J.J. (2010). Physiology and behavior of pigs before and after castration: Effects of two topical anesthetics. Animals.

[10] Trevisan C., Montillo M., Prandi A., Mkupasi E.M., Ngowi H.A., Johansen M.V. (2017). Hair cortisol and dehydroepiandrosterone concentration in naturally Taenia solium infected pigs in Tanzania. Gen. Comp. Endocrinol..

[11] Bergamin C., Comin A., Corazzin M., Faustini M., Peric T., Scollo A., Gottardo F., Montillo M., Prandi A. (2019). Cortisol, DHEA, and Sexual Steroid Concentrations in Fattening Pigs’ Hair. Animals.

[12] Fels M., Rauterberg S., Schwennen C., Ligges U., Herbrandt S., Kemper N., Schmicke M., Fels M., Rauterberg S., Schwennen C. (2019). Cortisol/dehydroepiandrosterone ratio in saliva: Endocrine biomarker for chronic stress in pigs?. Livest. Sci..

[13] Morgan L., Itin-Shwartz B., Koren L., Meyer J.S., Matas D., Younis A., Novak S., Weizmann N., Rapaic O., Weissam A.A. (2019). Physiological and economic benefits of abandoning invasive surgical procedures and enhancing animal welfare in swine production. Sci. Rep..

[14] Heimbürge S., Kanitz E., Otten W. (2019). The use of hair cortisol for the assessment of stress in animals. Gen. Comp. Endocrinol..

[15] Qiao S., Li X., Zilioli S., Chen Z., Deng H., Pan J., Guo W. (2016). Hair Measurements of Cortisol, DHEA, and DHEA to Cortisol Ratio as Biomarkers of Chronic Stress among People Living with HIV in China: Known-Group Validation. PLoS ONE.

[16] Mesarčová L., Kottferova J., Skurkova L., Leskova L., Kmecova N. (2017). Analysis of cortisol in dog hair—A potential biomarker of chronic stress: A review. Veterinární Med..

[17] Sharpley C.F., McFarlane J., Slominski A. (2011). Stress-linked cortisol concentrations in hair: What we know and what we need to know. Rev. Neurosci..

[18] Gow R., Thomson S., Rieder M., Van Uum S., Koren G. (2010). An assessment of cortisol analysis in hair and its clinical applications. Forensic Sci. Int..

[19] Macbeth B.J., Cattet M., Stenhouse G.B., Gibeau M.L., Janz D.M. (2010). Hair cortisol concentration as a non-invasive measure of long-term stress in free-ranging brown bears (Ursus arctos): Considerations with implications for other wildlife. Can. J. Zool..

[20] Casal N., Manteca X., Peña R.L., Bassols A., Fàbrega E. (2017). Analysis of cortisol in hair samples as an indicator of stress in pigs. J. Veter. Behav..

[21] Martelli G., Sardi L., Stancampiano L., Govoni N., Zannoni A., Nannoni E., Forni M., Bacci M.L. (2014). A study of some welfare-related parameters of hDAF transgenic pigs when compared with their conventional close relatives. Animals.

[22] Carroll G.A., Boyle L.A., Hanlon A., Palmer M.A., Collins L., Griffin K., Armstrong D., O’Connell N.E. (2018). Identifying physiological measures of lifetime welfare status in pigs: Exploring the usefulness of haptoglobin, C-reactive protein and hair cortisol sampled at the time of slaughter. Ir. Veter. J..

[23] Roelofs S., Godding L., de Haan J.R., van der Staay F.J., Nordquist R. (2019). Effects of parity and litter size on cortisol measures in commercially housed sows and their offspring. Physiol. Behav..

[24] Otten W., Heimbürge S., Kanitz E., Tuchscherer A. (2020). It’s getting hairy—External contamination may affect the validity of hair cortisol as an indicator of stress in pigs and cattle. Gen. Comp. Endocrinol..

[25] Davenport M.D., Tiefenbacher S., Lutz C.K., Novak M.A., Meyer J.S. (2006). Analysis of endogenous cortisol concentrations in the hair of rhesus macaques. Gen. Comp. Endocrinol..

[26] Kroshko T., Kapronczai L., Cattet M.R.L., Macbeth B.J., Stenhouse G.B., Obbard M.E., Janz D.M. (2017). Comparison of methanol and isopropanol as wash solvents for determination of hair cortisol concentration in grizzly bears and polar bears. MethodsX.

[27] Rantzer D., Svendsen J. (2010). Slatted versus solid floors in the dung area of farrowing pens: Effects on hygiene and pig performance, birth to weaning. Acta. Agric. Scand. A.

[28] Tsanaclis L., Wicks J.F. (2008). Differentiation between drug use and environmental contamination when testing for drugs in hair. Forensic Sci. Int..

[29] Seddon Y., Guy J., Edwards S. (2012). Optimising oral fluid collection from groups of pigs: Effect of housing system and provision of ropes. Veter. J..

[30] IUPAC-IUB (1989). IUPAC-IUB joint commission on biochemical nomenclature. The nomenclature of steroids. Recommendations. Eur. J. Biochem..

[31] Gonzalo-Lumbreras R., Izquierdo-Hornillos R. (2003). Method development for corticosteroids and anabolic steroids by micellar liquid chromatography. J. Chromatogr. B.

[32] Eser H.P., Pötsch L., Skopp G., Moeller M.R. (1997). Influence of sample preparation on analytical results: Drug analysis [GC/MS] on hair snippets versus hair powder using various extraction methods. Forensic Sci. Int..

[33] Kidwell D.A., Lee E.H., DeLauder S.F. (2000). Evidence for bias in hair testing and procedures to correct bias. Forensic Sci. Int..

[34] Hamel A.F., Meyer J.S., Henchey E., Dettmer A.M., Suomi S.J., Novak M.A. (2011). Effects of shampoo and water washing on hair cortisol concentrations. Clin. Chim. Acta.

[35] Wang X., Busch J.R., Banner J., Linnet K., Johansen S.S. (2019). Hair testing for cortisol by UPLC–MS/MS in a family: External cross-contamination from use of cortisol cream. Forensic Sci. Int..

[36] Carlsson H.-E., Lyberg K., Royo F., Hau J. (2007). Quantification of stress sensitive markers in single fecal samples do not accurately predict excretion of these in the pig. Res. Veter. Sci..

[37] Gerace E., Veronesi A., Martra G., Salomone A., Vincenti M. (2017). Study of cocaine incorporation in hair damaged by cosmetic treatments. Forensic Chem..

